# Immune Escape Associated with RBD Omicron Mutations and SARS-CoV-2 Evolution Dynamics

**DOI:** 10.3390/v14081603

**Published:** 2022-07-22

**Authors:** Aleksandr V. Kudriavtsev, Anna V. Vakhrusheva, Valery N. Novoseletsky, Marine E. Bozdaganyan, Konstantin V. Shaitan, Mikhail P. Kirpichnikov, Olga S. Sokolova

**Affiliations:** 1 Faculty of Biology, Lomonosov Moscow State University, 119234 Moscow, Russia; kudrjavcev.aleks.2011@post.bio.msu.ru (A.V.K.); vakhrusheva.anna.2017@post.bio.msu.ru (A.V.V.); valery.novoseletsky@yandex.ru (V.N.N.); bozdaganyan@mail.bio.msu.ru (M.E.B.); k.v.shaitan@molsim.org (K.V.S.); kirpichnikov@inbox.ru (M.P.K.); 2 Faculty of Biology, Shenzhen MSU-BIT University, Shenzhen 518172, China; 3 N. Semenov Federal Research Center for Chemical Physics, Russian Academy of Sciences, 119991 Moscow, Russia

**Keywords:** SARS-CoV-2, COVID-19, Omicron, bioinformatics, immune escape, RBD mutations, vaccine development

## Abstract

The evolution and the emergence of new mutations of viruses affect their transmissibility and/or pathogenicity features, depending on different evolutionary scenarios of virus adaptation to the host. A typical trade-off scenario of SARS-CoV-2 evolution has been proposed, which leads to the appearance of an Omicron strain with lowered lethality, yet enhanced transmissibility. This direction of evolution might be partly explained by virus adaptation to therapeutic agents and enhanced escape from vaccine-induced and natural immunity formed by other SARS-CoV-2 strains. Omicron’s high mutation rate in the Spike protein, as well as its previously described high genome mutation rate (Kandeel et al., 2021), revealed a gap between it and other SARS-CoV-2 strains, indicating the absence of a transitional evolutionary form to the Omicron strain. Therefore, Omicron has emerged as a new serotype divergent from the evolutionary lineage of other SARS-CoV-2 strains. Omicron is a rapidly evolving variant of high concern, whose new subvariants continue to manifest. Its further understanding and the further monitoring of key mutations that provide virus immune escape and/or high affinity towards the receptor could be useful for vaccine and therapeutic development in order to control the evolutionary direction of the COVID-19 pandemic.

## 1. Introduction

SARS-CoV-2 is a virulent and pathogenic virus that caused a global pandemic in late 2019, which is still ongoing. Currently (May 2022), about 515 million cases and 6.2 million deaths have been registered [1]. Coronaviruses are enveloped viruses that possess a protein nucleocapsid and a lipid supercapsid with embedded surface proteins. The most studied surface protein is the Spike or S-protein, which is used by the virus to recognize and bind the ACE2 receptor [2,3]. After binding, the S-protein undergoes cleavage by host proteases at the furin site between the S1 and S2 subunits [4]. The S1 subunit remains bound to the ACE2 receptor [5], and S2 undergoes significant conformational changes upon transitioning to the post-fusion state [6]. In this state, it binds to the surface of the host cell, allowing fusing of the viral and cell membranes [7].

**Figure 1:**
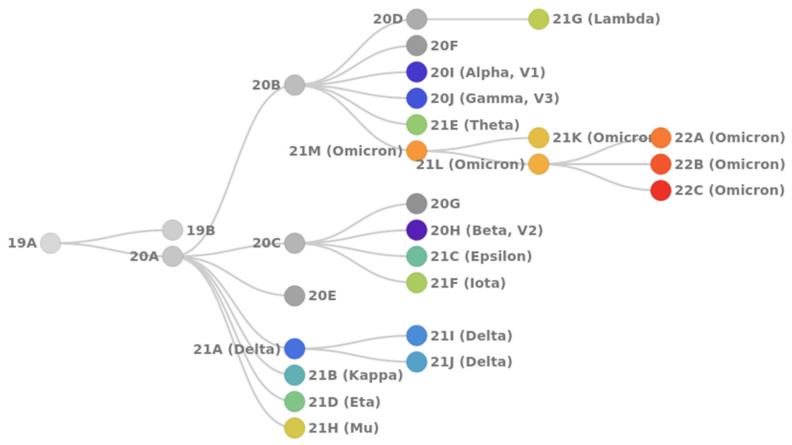
Phylogenetic relationships of existing SARS-CoV-2 clades. Clades 19A and 19B are ancestor lineages that emerged in Wuhan. Clade 20A emerged from 19A and was dominant during the European outbreak in March 2020. Clades 20B and 20C are large genetically distinct subclades from 20A that emerged in early 2020. Clades from 20D to 20J emerged over the summer of 2020 and include three Variants of Concern (VoC) [9]: Alpha (lineage B.1.1.7), Beta (lineage B.1.351), Gamma (lineage P.1). Clades from 21A to 21J include the VoC Delta and several Variants of Interest (VoI)—Lambda (lineage C.37), Mu (lineage B.1.621), Epsilon (lineages B.1.429), and some others. Clades 21K (BA.1) and 21L (BA.2) Omicron sublineages emerged from the South Africa strain 21M (lineage B.1.1.529); clades 22A, 22B, and 22C are the currently circulating sublineages of 21L Omicron. Generated by Nextstrain [10,11].

According to the NextClade classification [8], the following SARS-CoV-2 clades can be distinguished (Figure 1).

By May 2022, five SARS-CoV-2 Variants of Concern (VoC) with global public health affect have been registered worldwide:

1. 20I Alpha (B.1.1.7, V1): first VoC described in the United Kingdom in late December 2020;
2. 20H Beta (B.1.351, V2): first VoC reported in South Africa in December 2020;
3. 20J Gamma (P.1, V3): first reported in Brazil in early January 2021;
4. 21A Delta (B.1.617.2): first reported in India in December 2020; includes sublineages 21I and 21J;
5. 21M Omicron (B.1.1.529): first reported in South Africa in November 2021; includes two sublineages: 21K (BA.1) and 21L (BA.2).

**Figure 2:**
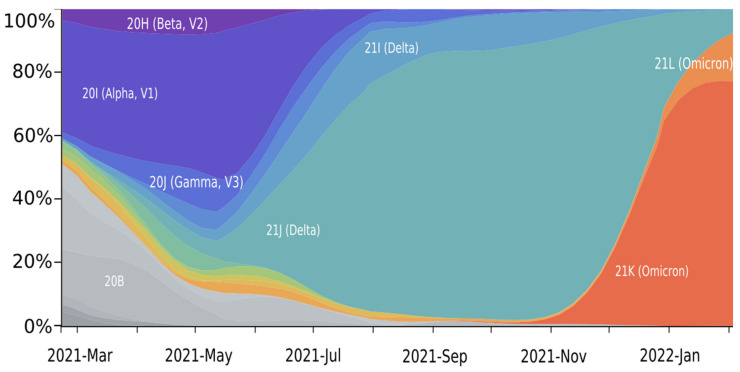
SARS-CoV-2 clade distribution dynamics. Colors are allocated according to Nexstrain Clades: 20B—grey; 20H—purple; 20I—dark blue; 20J—blue; 21A—light blue; 21I—lilac blue; 21J—blue-green; 21L—orange; 21K—orange red. Generated by NextStrain [10,11].

Using the web-based NextStrain toolkit [10], we analyzed the spread of SARS-CoV-2 based on 3257 SARS-CoV-2 genomes, sampled between February 2019 and February 2022 from GISAID [10,11] (Figure 2).

The worldwide spread of SARS-CoV-2 possesses a classical wave-like appearance. At the beginning of 2021, no single strain of SARS-CoV-2 was dominant, while several strains were distributed simultaneously: 20B, 20H, and 20I. By mid-2021, the SARS-CoV-2 Delta strain began to spread and quickly displaced the previous strains. This strain includes two sublines: 21J (up to 87% representation) and 21I (up to 13% representation). From November 2021, Delta was displaced rapidly by the Omicron strain. The latter was represented in February 2022 at a ratio of 78% (21K sublineage) and 15% (21L sublineage). By 2022, the Delta strain remained in only 6% of sequenced samples, predominantly as the 21J sublineage.

## 2. Mutation Dynamic Is Associated with Omicron Evolution

**Figure 3:**
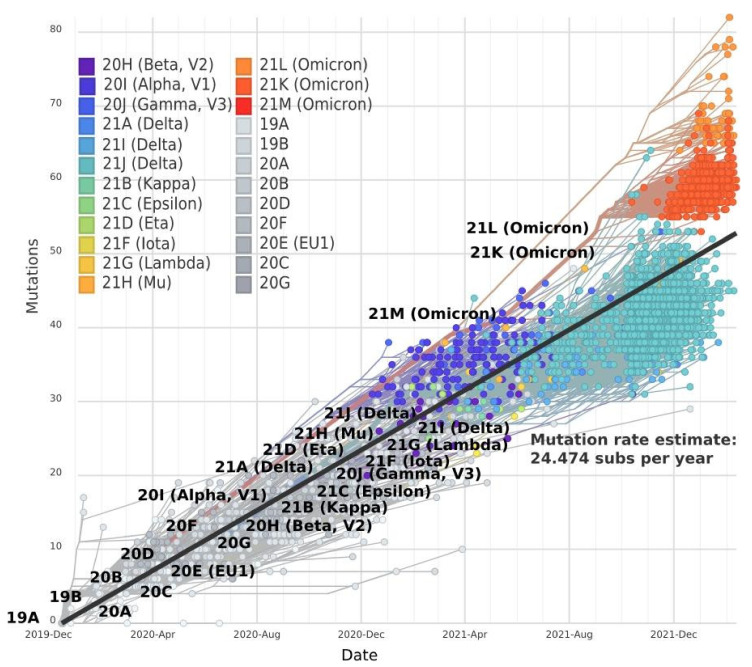
Time dependence of the accumulation rate of mutations in the entire SARS-CoV-2 genome constructed by NextStrain.org, according to GISAID data. Branches in phylogenetic trees were set according to NextClade classification [16]. Generated by NextStrain [10,11].

Since the beginning of the COVID-19 pandemic, the evolution of SARS-CoV-2 has moved in a linear regression trend (Figure 3). Between December 2019 and October 2020, about two mutations per month were registered [12,13]. By July 2022, 29,713 SNPs (single-nucleotide polymorphisms) were identified within the entire SARS-CoV-2 genome according to The National Genomics Data Center (NGDC), a section of the China National Center for Bioinformation (CNCB) [14]. The average mutation rate in SARS-CoV-2 is now approximately 24.5 mutations per year. Beta (20H), Iota (21F), and Epsilon (21C) strains have a similar divergence of 19–21 mutations from the common Wuhan ancestor 19A. Delta sublineages (21A and 21J) differ from 19A by 20 and 31 mutations, respectively.

Notably, there was an acceleration of evolution with the emergence of the Omicron strain. Despite the significant difference in Delta and Omicron strains, the divergence for the mutant Delta strains 21I and 21J from the ancestor strain 19A was as much as 35 and 44 mutations, respectively. The divergence of Omicron sublineages (21K and 21L) from the parental lineage has reached 52 and 78 mutations, respectively. Thus, Omicron has accumulated the largest number of new mutations [15] and possess the highest mutation rate (Figure 3).

Based on the mutation rate, different directions of SARS-CoV-2 strain evolution can be highlighted. The first direction of evolution includes the Delta strains derived from line 21A, which possess a slower mutation rate. The second direction is the group derived from line 20B, including the fast-evolving Omicron strain.

**Figure 4:**
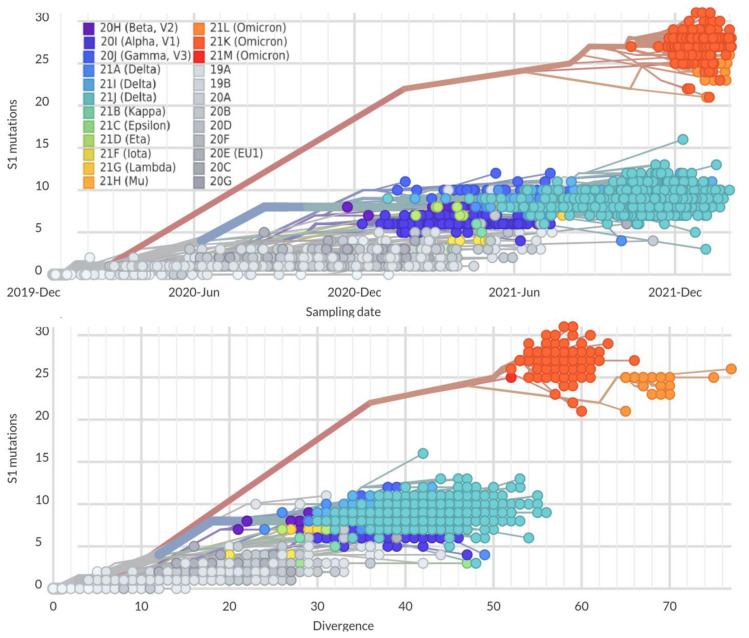
Dependence of mutation number in the S1 part of the Spike protein from sampling date (top) and divergence (total number of mutations, bottom) in genomes of different SARS-CoV-2 strains; the color code corresponds with different Nexstrain Clades. The analyzed data set was obtained by applying the parameters: “ncov gisaid global” [17]. Generated by NextStrain [10,11].

More precisely, the highest mutation rate was observed in the sequence of the S1-subunit of the SARS-CoV-2 Spike protein. This is one of the key regions that promote virus adaptation to humans. Until October 2021, the mutation rate in the S1-subunit reached five mutations per year in the mainstream strains (Alpha, Beta, Delta). By the end of 2021, the mutation rate reached 12–15 mutations/year. Starting with Omicron and its rapid evolution, a gap in the accumulation rate of new mutations in the S1 subunit emerged. It increased to 20–30 mutations/year in the S1 subunit, which is exhibits a fast level of evolution (Figure 4) and characterizes the SARS-CoV-2 adaptation mechanism.

This observable gap (43–63 nucleotides) indicates the possible absence of transitional variants between earlier SARS-CoV-2 strains and Omicron. Various evolutionary models suggest that the Omicron strain significantly differs from other variants and has been formed as a distant monophyletic class [17,18]. Some authors suppose that Omicron appeared as a result of high selection pressure and a “hypermutation” process affecting the S1-subunit and RBD as the main targets for neutralizing antibodies and preferable parts for Darwinian evolution for the development of immune escape [19]. On the other hand, there is evidence that the Omicron ancestor might have passed from humans to mice, acquired favorable mutations to infect this host, then passed back to humans [20]. Alternatively, it was shown that the Wuhan strain binds with a good specificity to cat ACE2, but not to mouse ACE2. Considering the fact that the Omicron strain efficiently binds both the murine and the human ACE2-receptors, the possible evolution pathway could be the following: human–cat–mouse–human [21]. Thus, an inter-species evolutionary trajectory for the Omicron outbreak is possible [20,21]. In addition, immunocompromised HIV-infected patients could be favorable hosts for SARS-CoV-2 evolution [22,23].

It is worth noting that significant Omicron changes in the reduced virulence and, thereafter, affinity towards the Syrian hamsters [24] might be an important issue for translational medicine studies, as COVID-19 experiments on Syrian hamsters are stated as the standard model.

## 3. Omicron Mutations Are Significant for SARS-CoV-2 Evolution

Most of the emerging mutations have little to no effect on the properties of the virus [25]. However, some mutations can significantly affect the pathogenicity, infectivity, transmissibility, and antigenicity of the virus. Some important mutations that facilitate the furin-mediated cleavage of the Spike protein and accelerate the process of virus–cell fusion are found outside of the S1-subunit. For instance, P681R is present in the Alpha, Delta, and Omicron strains [26]. Another key mutation, D614G, provides an increased affinity for the ACE2 receptor, which stimulates the infectivity of the virus and its transmission ability [27,28,29]. This mutation is located in the vicinity of the furin cleavage site, which leads to the conversion of the open RBD conformation by improving proteolysis at the S1/S2 site and increasing fusion between the membrane and the ACE2 receptor. It should be noted that this mutation occurred independently several times in the population [30,31], representing a convergent evolution path. This mutation does not affect the ability of the neutralizing antibodies to recognize the viral antigen [32,33,34]. However, it affects transmissibility, as the basic reproduction number (R0) was increased from 3.1 (614D) to 4.0 (614G) [29]. The structure of the Spike protein with a D614G mutation has been solved using cryo-electron microscopy [35,36,37].

Meanwhile, the majority of significant mutations for virus evolution are found in the RBD. The most frequent mutations found in currently circulated SARS-CoV-2 strains are summarized in Table 1.

The following positions were studied recently: 417, 452, 478, 484, and 501, demonstrating a high mutation rate. The N501Y mutation is widespread and is presented in Alpha, Beta, Gamma, and Omicron strains. The structure of the N501Y SARS-CoV-2 Spike-protein in complex with the ACE2 receptor was determined using cryo-electron microscopy with a resolution of 2.9 Å. The N501 amino acid residue was identified to be important for binding to the ACE2 receptor by forming an N501–G352 hydrogen bond and hydrophobically stacking the following amino acid residues: Tyr41(ACE2)–Lys353(ACE2)–Tyr505 [38,39]. Y501 has been shown to be introduced into the cavity of the ACE2 binding interface near the Y41 residue, resulting in the formation of an additional bond and an increase in the affinity of the ACE2 receptor for the N501Y mutant and its infectivity. However, this mutation did not significantly affect the structure, leaving important neutralizing epitopes in RBD available for antibodies [39].

Frequent mutations E484K/E484Q and L452R also allow RBD binding to the ACE2. Studies indicate that these mutations help avoid recognition by antibodies against the original coronavirus strain [40,41,42]. Deep mutagenesis has identified the E484 mutation as the most significant, which reduces neutralizing titers by an order of magnitude [41]. The presence of this mutation led to an almost complete absence of neutralization by monoclonal antibodies C121 and C144 [43] and convalescent plasma [40,44]. L452R appeared independently in several lineages of SARS-CoV-2 in December 2020 and February 2021, indicating the importance of this mutation for the adaptation of the virus to the increasing immune response of the population [42]. The L452R mutation enhanced binding affinity, transmissibility, fitness, and infectivity of SARS-CoV-2 [42]. Due to the unique T478K mutation, the Delta strain was relatively resistant to neutralizing antibodies after vaccination [45]. Double mutations T478K and L452R in the Delta strain increased the attraction between side chains of ACE2–E37 and RBD–R403, thereby increasing the affinity of the virus for the host receptor [46]. Molecular dynamic simulation showed that a triad mutation: E484K, K417N, and N501Y, induces conformational change to a greater extent, compared to the N501Y mutant alone, and potentially results in an escape mutant [47].

**Figure 5:**
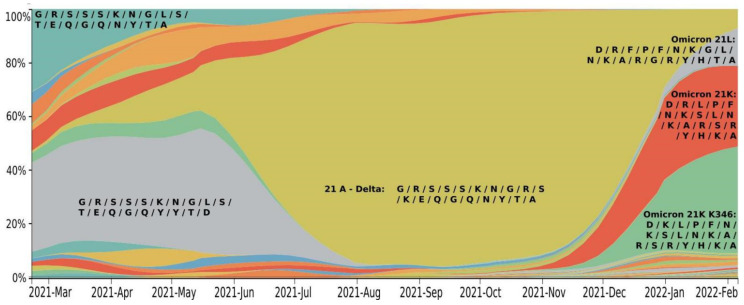
Time series of important mutations in the RBD of Spike. Generated by NextStrain [10,11]. Amino acid replacements in the RBD sequence at key positions (339/346/371/373/375/417/440/446/452/477/478/484/493/496/498/501/505/547/570) and their frequency in SARS-CoV-2 sublineages: 21A (Delta), 21K (Omicron BA.1), 21K (BA.1 + K346), 21L (Omicron BA.2) from February 2021 to February 2022. Letters indicate amino acids in the same order as the positions were listed above. Colors were allocated according to the different sets of amino acid mutations in the RBD: 21A—mustard; 21L—grey; 21K—orange red; 21K (BA.1 + K346)—green.

Based on our analysis of the mutation distribution, we suggest that key RBD mutations (Table 1) may be used to predict the behavior of the new strains. In this respect, several sublineages of the Delta strain widespread at the end of 2021 possess somewhat similar RBD sequences to previous strands (Figure 5) and underwent neutralization by natural- and vaccine-induced immunity. On the other hand, the sublineages of the Omicron strain, which replaced Delta in 2022, can escape immunity. Moreover, several Omicron sublineages (21L and 21K) were able to spread freely and cause new waves of the disease. Here the significant difference in the RBD of different Omicron sublineages and other SARS-CoV-2 strains can be detected. Classification based on frequent RBD mutations allowed us to isolate a new independent sublineage that differs from the 21K, called 21K + K346.

Thus, monitoring the sublineages based on frequent RBD mutations could be useful for further control of SARS-CoV-2 and vaccine renewal. Based on the RBD difference in key mutation positions, currently circulated strains can be classified into four main subgroups: 21A Delta, Omicron 21K (BA.1), Omicron 21K + K346 (BA.1), and Omicron 21L (BA.2) (Figure 5).

## 4. Omicron Is Characterized by High Immune Evasion

Since most of the antibodies are directed at the Spike protein, they appear to target RBD. Omicron, due to its specific mutations in RBD, successfully escapes most therapeutic antibodies (e.g., REGN10933, REGN10987, COV2-2196, COV2-2130, LY-CoV555, LY-CoV16, CT-P59). Only therapeutic antibodies of class 3 (S309) and 4 (A3, CR3022, S2A4, S304, S2X35, H014, COVA1-16, S2X259, and DH1047) targeting regions that do not directly interact with ACE2, but interfere with its binding, can potently neutralize Omicron with some efficiency [48,49,50].

Antibodies of recovered individuals who have been infected with previous COVID-19 strains or immunized with vaccines based on the Wuhan antigen also cannot efficiently neutralize Omicron [15]. A pseudovirus assay demonstrated that Omicron virus neutralization by plasma of convalescent or twice-vaccinated people (with BNT162b2, mRNA-1273, ChAdOx1, Ad26.COV2.S, Sputnik V, BBIBP-CorV) was significantly reduced or absent in comparison with the ancestral strain [51,52,53,54,55]. A decrease in GMT (geometric mean neutralization antibody titers) against Omicron was registered from 20-fold to more than 100-fold in contrast to the Wuhan-Hu-1 antigen [51,52,53,54,55]. Similar results were obtained in the live virus neutralization assay [56,57]. Moreover, only ~20% of BNT162b2 recipients had some detectable neutralizing levels of antibodies against Omicron, whereas none of the Sinovac recipients had neutralizing antibody titer against any Omicron isolate [56].

VoC strains Alpha, Gamma, and Delta had a similar and less discernible immunity escape. The Alpha variant undergoes neutralization by some monoclonal antibodies targeting the NTD of the Spike protein [58]; given the immunodominance of RBD, the overall decrease in the neutralizing antibodies produced by natural infection [58] and vaccines BNT162b2, mRNA-1273 [58], ChAdOx1 nCoV-19 [59], BBIBP-CorV [60], and Covaxin [61] is insignificant (2–3-fold reduction) and is predominantly preserved.

The Gamma variant has a moderate decrease (4.5–6.7-fold) in neutralization with post-vaccination sera after two doses of BNT162b2 and mRNA-1273 [62]. Convalescent sera from patients infected with Beta and Gamma strains, as well as sera from people vaccinated by BNT162b2 and ChAdOx1 nCoV-19, had lower, but not tremendous, neutralization to Delta sublineages, in comparison with the Wuhan reference. The average reduction of GMT was in the range of 2.2–4.3-fold [52,63,64]. Therefore, there is no widespread escape from neutralization by the Alpha, Gamma, and Delta strains.

Presumably, the resulting combination of K417N and E484K mutations, as well as changes in NTD, allow the Beta strain to significantly bypass the response of polyclonal antibodies, compared with the Wuhan reference. They escape both RBD- and NTD-specific antibodies [43,58,65,66,67] from COVID-19 convalescent plasma (11–33-fold), as well as from vaccine-induced antibodies: mRNA-1273 (19.2–27.7-fold), BNT162b2 (6.5–42.4-fold) [43,52,58,62], ChaAdOx1 [68], BBIBP-CorV [60], and NVX-CoV2373 [69,70].

The plasma of COVID-19 patients infected with strains advancing Omicron and plasma from vaccinated patients demonstrates reductions in neutralization activity of no more than 10-fold, except for the Beta strain, which has a 10–40-fold reduction. On the other hand, the decrease in neutralization activity against Omicron was 20–80-fold, in some cases up to 120-fold. Thus, the Beta strain escapes neutralization more strongly than Alpha, Gamma, and Delta strains, but less than Omicron.

This VoC immunity escape data are in accordance with the quantity of significant RBD mutations that provide escape from neutralizing antibodies. In Table 2, the most significant RBD mutations responsible for immune escape are highlighted in regard to every VoC strain. The Alpha strain has the least resistance to neutralization and has only one mutation (N501Y) associated with immune escape. Gamma and Delta strains have similar reductions in the neutralization level, each possessing three different RBD mutations: Gamma—K417N, E484K, N501Y; Delta—E484Q, L452R, T478K (Table 2). Despite the presence of the same triplet of mutations as in Gamma (E484K, K417N/T, N501Y), the Beta strain was shown to be more resistant to convalescent and vaccine samples [71], presumably due to additional changes outside of the RBD. Beta and Omicron strains have the highest potency to escape natural or vaccine-based immunity and share similar K417/E484/N501 triad mutations. Omicron has four significant RBD mutations (the highest number from all VoC) that markedly influence immune escape: the above-mentioned triad mutations and T478K, previously presented in the Delta strain.

Based on cross-neutralization experiments among patients infected with the ancestral, Alpha, Beta, Gamma, and Delta variants, as well as those vaccinated with two consequent Moderna, Pfizer/BioNTech, or AstraZeneca vaccines, it was demonstrated that Alpha, Beta, Gamma, and Delta strains belong to one antigenic cluster, while Omicron represents a separate antigenic variant [72]. Therefore, Omicron can represent a different serotype from other SARS-CoV-2 strains and, thus, Omicron-stimulated immunity cannot protect against other strains as well as immunity from other strains that demonstrated Omicron-negligible neutralization [73].

## 5. Omicron Has Achieved Balance between High Transmissibility and Low Mortality

The highest level of Omicron immune escape in comparison with other SARS-CoV-2 variants might also be a sign of high transmissibility, but low mortality characteristics [74]. This is in concordance with the fact that virus evolution aims to adapt to the host and achieve evolutionary stasis in long-term virus–host relationships [75]. In the case of SARS-CoV-2, it is expressed in the adaptation of the S1-subunit, especially RBD, to the ACE2-receptor.

Three possible scenarios of virus evolution exist: (i) increased virulence; (ii) unchanged virulence, and (iii) decreased virulence; the latter scenario is more common than the first two [76]. Virulence is often described in terms of transmission rate and host mortality rate [77]. Typical trade-off models for virus evolution state that mortality and transmission rates would have optimal proportions achieved during adaptation [77]. In other words, host mortality limits transmission.

**Figure 6:**
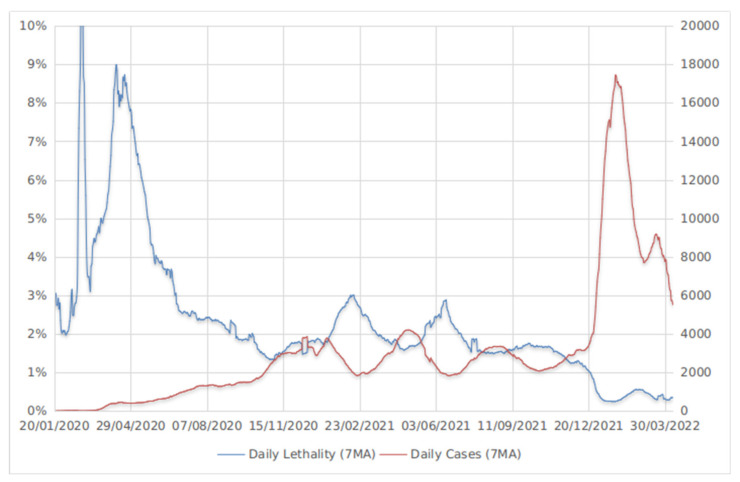
Fluctuations of a 7-day moving average for daily lethality and daily COVID-19 cases; data obtained from [78].

It is important to note that since the start of the COVID-19 pandemic, virus mortality and transmissibility characteristics have been unbalanced. In the first half of 2020, daily cases rose from zero to several thousand per day, and mortality rates were as high as 9 to 10%. A balance between daily cases and daily lethality was achieved in November 2020, when the mortality rate fluctuated around 1.5–3% and transmissibility was in the same range of 2–4 thousand cases. By the end of 2021, the emergence of Omicron disturbed this balance: daily lethality has declined and fluctuated at around 0.5%, and new daily cases skyrocketed to about 10–18 thousand per day. Therefore, Omicron adaptation to the host made it possess low lethality and increased transmissibility features. The hypothesis of a balance between mortality and transmission of the SARS-CoV-2 virus is supported by epidemiological data (Figure 6).

A possible reason for the high transmissibility of Omicron is the repeated rounds of selection and the emergence of beneficial RBD mutations providing immune escape [19]. This is supported by the possible emergence of Omicron in immunocompromised HIV-infected patients [22,23]. Another hypothesis is the selective pressure of vaccination and influence of Spike antigenic sites. Widespread vaccination could have led to the evolution of SARS-CoV-2 in the direction of immunity escape and high transmissibility, but not in the direction of increasing mortality [79].

The SARS-CoV-2 balance between transmissibility and lethality is expected to remain in the future. In the long term, a natural decline in lethality and an increase in the incidence of SARS-CoV-2 with the emergence of new strains is possible. This trend will determine the further evolution of SARS-CoV-2, bringing it closer to typical seasonal influenza arising from other types of coronaviruses (HCoV 229E, NL63, OC43, and HKU1), which are currently responsible for 10% to 30% of adult upper respiratory tract infections [80].

Since pathogenicity features are based on various evolutionary scenarios of virus adaptation to the host, analysis of the evolutionary direction and monitoring of key mutations associated with main virus characteristics may be useful for further strategies to optimize anti-COVID-19 vaccines in polyvalent formations and other therapeutic developments.

## 6. Discussion

The origin of the Omicron variant of SARS-CoV-2 remains unclear and requires further investigation. Here, we described the four main currently circulating Omicron sublineages (21A, 21L, 21K, 21K + K346) based on the dynamic distribution analysis of key RBD mutations. A lower immune response and significant ability to escape immunity may lead to lower mortality and higher transmissibility of all Omicron sublineages. Oppositely, enormous dysregulation and a strong immune response may lead to the development of systemic inflammatory response syndrome (SIRS) and to higher mortality [81]. Therefore, the fast evolution of Omicron may be provoked by the utilization of different therapeutics, such as vaccines and monoclonal neutralizing antibodies, for the treatment of the patient.

The rapid emergence of new mutations in the Spike protein of the virus affects its virulence and its avoidance of the developed immune defense. Examining the mutation effect on the neutralizing properties of vaccines and antibodies is important for the disclosure of immunodominant regions [82] and for the development and improvement of therapies against SARS-CoV-2.

It is likely that SARS-CoV-2 will continue its evolution and may lead to the emergence of variants of unpredictable severity [83]. This is the rare situation in which vaccination led to the formation of more hazardous types of viruses [84]. Typically, vaccination evolves another virus property—immune escape. Another hypothesis is that frequent vaccine renewal that reduces pathogen virulence helps lower the possibility of a more hazardous strain. The virus may evolve either in the direction of immunity escape or in the direction of virulence increase, but not in both directions simultaneously [79]. Since most vaccines only immunize against the Spike protein, evolution of escape may be easier against vaccine immunity than against natural immunity.

One might assume that vaccine optimization in accordance with the above-mentioned RBD mutations in Omicron would provide immunity against it. However, the Omicron-based vaccine and boosterization evoked an impaired serologic response and the emergence of neutralization antibodies against Omicron and wild-type SARS-CoV-2, in comparison with vaccines based on the Wuhan antigen [85].

In contrast, fully-vaccinated people with either homologous BNT162b2 vaccination or heterologous ChAdOx1-S–BNT162b2 vaccines based on the Wuhan strain, as well as convalescent-vaccinated patients, have some cross-neutralization antibodies against Omicron [86]. Thus, booster immunization by vaccines significantly increases the neutralization level titer against Omicron [53,54,55,87,88,89,90,91,92,93].

This can be explained by the fact that Omicron stimulates very specific immune responses and represents a distinct antigenic serotype. Therefore, vaccine boosters based on the Omicron strain are less efficient than those based on ancestral one.

Yet, polyvalent vaccines should also be considered as an option. Recent studies of the polyvalent vaccine from Moderna based on Wuhan and Beta strains as boosters showed more pronounced antibody titers against all SARS-CoV-2 strains, in comparison with the monovalent Wuhan-booster [94]. As we have demonstrated, sequences of RBD are different in Delta (21A) and Omicron sublineages, which suggests the need for different antigens in vaccines against these forms of SARS-CoV-2.

Under this scenario, future vaccination programs should be planned seasonally rather than on a regular basis, as is currently the case with the influenza virus. Optimally, a one-shot vaccine against both influenza and COVID-19 should become available, such developments are underway [95].

## Figures and Tables

**Table 1:** The key RBD mutations in the SARS-CoV-2 Spike protein. The assignments to Delta (21A) and Omicron strains (21K and 21L) are shown.

		21L BA.2	21K K346	Title 3 BA.1	21A (Delta)
Prevalence	1 January 2022	3%	26%	31%	29%
	6 February 2022	14%	40%	30%	7%
RBD mutations	339	D	D	D	G
	346	R	K	R	R
	371	F	L	L	S
	373	P	P	P	S
	375	F	F	F	S
	417	N	N	N	K
	440	K	K	K	N
	446	G	S	S	G
	452	L	L	L	R
	477	N	N	N	S
	478	K	K	K	K
	484	A	A	A	E
	493	R	R	R	Q
	496	G	S	S	G
	498	R	R	R	Q
	501	Y	Y	Y	N
	505	H	H	H	Y
	547	T	K	K	T

**Table 2:** The most significant mutations providing immune escape of VoC strains. Number of pluses reveal the strength of the immune escape.

VoC	K417N	E484K	N501Y	L452R	T478K	Immune Escape
Alpha			+			+
Beta	+	+	+			+++++
Gamma	+ (K417T)	+	+			+++
Delta		+ (E484Q)		+	+	++
Omicron	+	+ (E484A)	+		+	+++++++

## References

[1] WHO Coronavirus (COVID-19) Dashboard. https://covid19.who.int/.

[2] Hoffmann M., Kleine-Weber H., Schroeder S., Krüger N., Herrler T., Erichsen S., Schiergens T.S., Herrler G., Wu N.-H., Nitsche A. (2020). SARS-CoV-2 cell entry depends on ACE2 and TMPRSS2 and is blocked by a clinically proven protease inhibitor. Cell.

[3] Wang S., Guo F., Liu K., Wang H., Rao S., Yang P., Jiang C. (2008). Endocytosis of the receptor-binding domain of SARS-CoV spike protein together with virus receptor ACE2. Virus Res..

[4] Belouzard S., Chu V.C., Whittaker G.R. (2009). Activation of the SARS coronavirus spike protein via sequential proteolytic cleavage at two distinct sites. Proc. Natl. Acad. Sci. USA.

[5] Song W., Gui M., Wang X., Xiang Y. (2018). Cryo-EM structure of the SARS coronavirus spike glycoprotein in complex with its host cell receptor ACE2. PLoS Pathog..

[6] Walls A.C., Tortorici M.A., Snijder J., Xiong X., Bosch B.-J., Rey F.A., Veesler D. (2017). Tectonic conformational changes of a coronavirus spike glycoprotein promote membrane fusion. Proc. Natl. Acad. Sci. USA.

[7] Li F. (2016). Structure, function, and evolution of coronavirus spike proteins. Annu. Rev. Virol..

[8] Clade Naming & Definitions-SARS-CoV-2 Workflow Documentation. https://docs.nextstrain.org/projects/ncov/en/latest/reference/naming_clades.html.

[9] Tracking SARS-CoV-2 Variants. https://www.who.int/activities/tracking-SARS-CoV-2-variants.

[10] Hadfield J., Megill C., Bell S.M., Huddleston J., Potter B., Callender C., Sagulenko P., Bedford T., Neher R.A. (2018). Nextstrain: Real-time tracking of pathogen evolution. Bioinformatics.

[11] Sagulenko P., Puller V., Neher R.A. (2018). TreeTime: Maximum-likelihood phylodynamic analysis. Virus Evol..

[12] Leung K., Shum M.H., Leung G.M., Lam T.T., Wu J.T. (2021). Early transmissibility assessment of the N501Y mutant strains of SARS-CoV-2 in the United Kingdom, October to November 2020. Eurosurveillance.

[13] Boehm E., Kronig I., Neher R.A., Eckerle I., Vetter P., Kaiser L. (2021). Novel SARS-CoV-2 variants: The pandemics within the pandemic. Clin. Microbiol. Infect..

[14] “RCoV19 Version 4.0 2019新型冠状病毒信息库.” RCoV19—2019新型冠状病毒信息库. https://ngdc.cncb.ac.cn/ncov/.

[15] Zhang X., Wu S., Wu B., Yang Q., Chen A., Li Y., Zhang Y., Pan T., Zhang H., He X. (2021). SARS-CoV-2 Omicron strain exhibits potent capabilities for immune evasion and viral entrance. Signal Transduct. Target. Ther..

[16] Casalino L., Gaieb Z., Goldsmith J.A., Hjorth C.K., Dommer A.C., Harbison A.M., Fogarty C.A., Barros E.P., Taylor B.C., McLellan J.S. (2020). Beyond Shielding: The Roles of Glycans in the SARS-CoV-2 Spike Protein. ACS Cent. Sci..

[17] Nextstrain. https://nextstrain.org/.

[18] Kandeel M., Mohamed M.E.M., Abd El-Lateef H.M., Venugopala K.N., El-Beltagi H.S. (2021). Omicron Variant Genome Evolution and Phylogenetics. J. Med. Virol..

[19] Berkhout B., Herrera-Carrillo E. (2022). SARS-CoV-2 Evolution: On the Sudden Appearance of the Omicron Variant. J. Virol..

[20] Wei C., Shan K.J., Wang W., Zhang S., Huan Q., Qian W. (2021). Evidence for a Mouse Origin of the SARS-CoV-2 Omicron Variant. J. Genet. Genom..

[21] Xu Y., Wu C., Cao X., Gu C., Liu H., Jiang M., Wang X., Yuan Q., Wu K., Liu J. (2022). Structural and biochemical mechanism for increased infectivity and immune evasion of Omicron BA.2 variant compared to BA.1 and their possible mouse origins. Cell Res..

[22] Tarcsai K.R., Corolciuc O., Tordai A., Ongrádi J. (2022). SARS-CoV-2 infection in HIV-infected patients: Potential role in the high mutational load of the Omicron variant emerging in South Africa. GeroScience.

[23] Hoffman S.A., Costales C., Sahoo M.K., Palanisamy S., Yamamoto F., Huang C., Verghese M., Solis D.A., Sibai M., Subramanian A. (2021). SARS-CoV-2 Neutralization Resistance Mutations in Patient with HIV/AIDS, California, USA. Emerg. Infect. Dis..

[24] Abdelnabi R., Foo C.S., Zhang X., Lemmens V., Maes P., Slechten B., Raymenants J., André E., Weynand B., Dallmeier K. (2022). The omicron (B.1.1.529) SARS-CoV-2 variant of concern does not readily infect Syrian hamsters. Antivir. Res..

[25] Frost S.D.W., Magalis B.R., Kosakovsky Pond S.L. (2018). Neutral Theory and Rapidly Evolving Viral Pathogens. Mol. Biol. Evol..

[26] Zhou B., Thao T.T.N., Hoffmann D., Taddeo A., Ebert N., Labroussaa F., Pohlmann A., King J., Steiner S., Kelly J.N. (2021). SARS-CoV-2 spike D614G change enhances replication and transmission. Nature.

[27] Hou Y.J., Chiba S., Halfmann P., Ehre C., Kuroda M., Dinnon K.H., Leist S.R., Schäfer A., Nakajima N., Takahashi K. (2020). SARS-CoV-2 D614G Variant Exhibits Efficient Replication Ex Vivo and Transmission in Vivo. Science.

[28] Yurkovetskiy L., Wang X., Pascal K.E., Tomkins-Tinch C., Nyalile T.P., Wang Y., Baum A., Diehl W.E., Dauphin A., Carbone C. (2020). Structural and Functional Analysis of the D614G SARS-CoV-2 Spike Protein Variant. Cell.

[29] Volz E., Hill V., McCrone J.T., Price A., Jorgensen D., O’Toole Á., Southgate J., Johnson R., Jackson B., Nascimento F.F. (2021). Evaluating the Effects of SARS-CoV-2 Spike Mutation D614G on Transmissibility and Pathogenicity. Cell.

[30] Zhan X.-Y., Zhang Y., Zhou X., Huang K., Qian Y., Leng Y., Yan L., Huang B., He Y. (2020). Molecular Evolution of SARS-CoV-2 Structural Genes: Evidence of Positive Selection in Spike Glycoprotein. bioRxiv.

[31] Korber B., Fischer W.M., Gnanakaran S., Yoon H., Theiler J., Abfalterer W., Hengartner N., Giorgi E.E., Bhattacharya T., Foley B. (2020). Tracking Changes in SARS-CoV-2 Spike: Evidence That D614G Increases Infectivity of the COVID-19 Virus. Cell.

[32] Ozono S., Zhang Y., Ode H., Sano K., Tan T.S., Imai K., Miyoshi K., Kishigami S., Ueno T., Iwatani Y. (2021). SARS-CoV-2 D614G Spike Mutation Increases Entry Efficiency with Enhanced ACE2-Binding Affinity. Nat. Commun..

[33] Plante J.A., Liu Y., Liu J., Xia H., Johnson B.A., Lokugamage K.G., Zhang X., Muruato A.E., Zou J., Fontes-Garfias C.R. (2020). Spike Mutation D614G Alters SARS-CoV-2 Fitness. Nature.

[34] Gobeil S.M., Janowska K., McDowell S., Mansouri K., Parks R., Manne K., Stalls V., Kopp M.F., Henderson R., Edwards R.J. (2021). D614G Mutation Alters SARS-CoV-2 Spike Conformation and Enhances Protease Cleavage at the S1/S2 Junction. Cell Rep..

[35] Benton D.J., Wrobel A.G., Roustan C., Borg A., Xu P., Martin S.R., Rosenthal P.B., Skehel J.J., Gamblin S.J. (2021). The Effect of the D614G Substitution on the Structure of the Spike Glycoprotein of SARS-CoV-2. Proc. Natl. Acad. Sci. USA.

[36] Verkhivker G.M., Agajanian S., Oztas D., Gupta G. (2021). Computational Analysis of Protein Stability and Allosteric Interaction Networks in Distinct Conformational Forms of the SARS-CoV-2 Spike D614G Mutant: Reconciling Functional Mechanisms through Allosteric Model of Spike Regulation. J. Biomol. Struct. Dyn..

[37] Saito A., Irie T., Suzuki R., Maemura T., Nasser H., Uriu K., Kosugi Y., Shirakawa K., Sadamasu K., Kimura I. (2021). SARS-CoV-2 Spike P681R Mutation Enhances and Accelerates Viral Fusion. bioRxiv.

[38] Wang Y., Liu M., Gao J. (2020). Enhanced Receptor Binding of SARS-CoV-2 through Networks of Hydrogen-Bonding and Hydrophobic Interactions. Proc. Natl. Acad. Sci. USA.

[39] Zhu X., Mannar D., Srivastava S.S., Berezuk A.M., Demers J.P., Saville J.W., Leopold K., Li W., Dimitrov D.S., Tuttle K.S. (2021). Cryo-Electron Microscopy Structures of the N501Y SARS-CoV-2 Spike Protein in Complex with ACE2 and 2 Potent Neutralizing Antibodies. PLoS Biol..

[40] Liu Z., VanBlargan L.A., Bloyet L.M., Rothlauf P.W., Chen R.E., Stumpf S., Zhao H., Errico J.M., Theel E.S., Liebeskind M.J. (2021). Identification of SARS-CoV-2 Spike Mutations That Attenuate Monoclonal and Serum Antibody Neutralization. Cell Host Microbe.

[41] Greaney A.J., Loes A.N., Crawford K.H.D., Starr T.N., Malone K.D., Chu H.Y., Bloom J.D. (2021). Comprehensive Mapping of Mutations to the SARS-CoV-2 Receptor-Binding Domain That Affect Recognition by Polyclonal Human Serum Antibodies. Cell Host Microbe.

[42] Tchesnokova V., Kulakesara H., Larson L., Bowers V., Rechkina E., Kisiela D., Sledneva Y., Choudhury D., Maslova I., Deng K. (2021). Acquisition of the L452R mutation in the ACE2-binding interface of Spike protein triggers recent massive expansion of SARS-Cov-2 variants. bioRxiv.

[43] Weisblum Y., Schmidt F., Zhang F., DaSilva J., Poston D., Lorenzi J.C., Muecksch F., Rutkowska M., Hoffmann H.H., Michailidis E. (2020). Escape from neutralizing antibodies by SARS-CoV-2 spike protein variants. Elife.

[44] Andreano E., Piccini G., Licastro D., Casalino L., Johnson N.V., Paciello I., Dal Monego S., Pantano E., Manganaro N., Manenti A. (2020). SARS-CoV-2 escape in vitro from a highly neutralizing COVID-19 convalescent plasma. bioRxiv.

[45] Wall E.C., Wu M., Harvey R., Kelly G., Warchal S., Sawyer C., Daniels R., Hobson P., Hatipoglu E., Ngai Y. (2021). Neutralising antibody activity against SARS-CoV-2 VOCs B.1.617.2 and B.1.351 by BNT162b2 vaccination. Lancet.

[46] Goher S.S., Ali F., Amin M. (2021). The Delta Variant Mutations in the Receptor Binding Domain of SARS-CoV-2 Show Enhanced Electrostatic Interactions with the ACE2. Med. Drug Discov..

[47] Nelson G., Buzko O., Spilman P., Niazi K., Rabizadeh S., Soon-Shiong P. (2021). Molecular Dynamic Simulation Reveals E484K Mutation Enhances Spike RBD-ACE2 Affinity and the Combination of E484K, K417N and N501Y Mutations (501Y.V2 Variant) Induces Conformational Change Greater than N501Y Mutant Alone, Potentially Resulting in an Escape Mutant. bioRxiv.

[48] McCallum M., Czudnochowski N., Rosen L.E., Zepeda S.K., Bowen J.E., Walls A.C., Hauser K., Joshi A., Stewart C., Dillen J.R. (2022). Structural basis of SARS-CoV-2 Omicron immune evasion and receptor engagement. Science.

[49] Nabel K.G., Clark S.A., Shankar S., Pan J., Clark L.E., Yang P., Coscia A., McKay L.G.A., Varnum H.H., Brusic V. (2022). Structural basis for continued antibody evasion by the SARS-CoV-2 receptor binding domain. Science.

[50] Li M., Lou F., Fan H. (2022). SARS-CoV-2 variant Omicron: Currently the most complete “escapee” from neutralization by antibodies and vaccines. Signal Transduct. Target. Ther..

[51] Cameroni E., Bowen J.E., Rosen L.E., Saliba C., Zepeda S.K., Culap K., Pinto D., VanBlargan L.A., De Marco A., di Iulio J. (2022). Broadly neutralizing antibodies overcome SARS-CoV-2 Omicron antigenic shift. Nature.

[52] Muik A., Lui B.G., Wallisch A.K., Bacher M., Mühl J., Reinholz J., Ozhelvaci O., Beckmann N., Güimil Garcia R.C., Poran A. (2022). Neutralization of SARS-CoV-2 Omicron by BNT162b2 mRNA vaccine-elicited human sera. Science.

[53] Doria-Rose N.A., Shen X., Schmidt S.D., O’Dell S., McDanal C., Feng W., Tong J., Eaton A., Maglinao M., Tang H. (2021). Booster of mRNA-1273 Strengthens SARS-CoV-2 Omicron Neutralization. medRxiv.

[54] Ai J., Zhang H., Zhang Y., Lin K., Zhang Y., Wu J., Wan Y., Huang Y., Song J., Fu Z. (2022). Omicron variant showed lower neutralizing sensitivity than other SARS-CoV-2 variants to immune sera elicited by vaccines after boost. Emerg. Microbes Infect..

[55] Schmidt F., Muecksch F., Weisblum Y., Da Silva J., Bednarski E., Cho A., Wang Z., Gaebler C., Caskey M., Nussenzweig M.C. (2022). Plasma Neutralization of the SARS-CoV-2 Omicron Variant. N. Engl. J. Med..

[56] Lu L., Mok B.W., Chen L.L., Chan J.M., Tsang O.T., Lam B.H., Chuang V.W., Chu A.W., Chan W.M., Ip J.D. (2021). Neutralization of SARS-CoV-2 Omicron variant by sera from BNT162b2 or Coronavac vaccine recipients. Clin. Infect. Dis..

[57] Cele S., Jackson L., Khoury D.S., Khan K., Moyo-Gwete T., Tegally H., San J.E., Cromer D., Scheepers C., Amoako D.G. (2022). Omicron extensively but incompletely escapes Pfizer BNT162b2 neutralization. Nature.

[58] Wang P., Nair M.S., Liu L., Iketani S., Luo Y., Guo Y., Wang M., Yu J., Zhang B., Kwong P.D. (2021). Antibody resistance of SARS-CoV-2 variants B.1.351 and B.1.1.7. Nature.

[59] Emary K.R.W., Golubchik T., Aley P.K., Ariani C.V., Angus B., Bibi S., Blane B., Bonsall D., Cicconi P., Charlton S. (2021). Efficacy of ChAdOx1 nCoV-19 (AZD1222) vaccine against SARS-CoV-2 variant of concern 202012/01 (B.1.1.7): An exploratory analysis of a randomised controlled trial. Lancet.

[60] Wang G.L., Wang Z.Y., Duan L.J., Meng Q.C., Jiang M.D., Cao J., Yao L., Zhu K.L., Cao W.C., Ma M.J. (2021). Susceptibility of Circulating SARS-CoV-2 Variants to Neutralization. N. Engl. J. Med..

[61] Sapkal G.N., Yadav P.D., Ella R., Deshpande G.R., Sahay R.R., Gupta N., Mohan V.K., Abraham P., Panda S., Bhargava B. (2021). Neutralization of UK-Variant VUI-202012/01 with COVAXIN Vaccinated Human Serum. bioRxiv.

[62] Garcia-Beltran W.F., Lam E.C., St Denis K., Nitido A.D., Garcia Z.H., Hauser B.M., Feldman J., Pavlovic M.N., Gregory D.J., Poznansky M.C. (2021). Multiple SARS-CoV-2 variants escape neutralization by vaccine-induced humoral immunity. Cell.

[63] Hu J., Wei X.Y., Xiang J., Peng P., Xu F.L., Wu K., Luo F.Y., Jin A.S., Fang L., Liu B.Z. (2021). Reduced neutralization of SARS-CoV-2 B.1.617 variant by convalescent and vaccinated sera. Genes Dis..

[64] Liu J., Liu Y., Xia H., Zou J., Weaver S.C., Swanson K.A., Cai H., Cutler M., Cooper D., Muik A. (2021). BNT162b2-elicited neutralization of B.1.617 and other SARS-CoV-2 variants. Nature.

[65] Wibmer C.K., Ayres F., Hermanus T., Madzivhandila M., Kgagudi P., Oosthuysen B., Lambson B.E., de Oliveira T., Vermeulen M., van der Berg K. (2021). SARS-CoV-2 501Y.V2 escapes neutralization by South African COVID-19 donor plasma. Nat. Med..

[66] Li Q., Nie J., Wu J., Zhang L., Ding R., Wang H., Zhang Y., Li T., Liu S., Zhang M. (2021). SARS-CoV-2 501Y.V2 variants lack higher infectivity but do have immune escape. Cell.

[67] Cele S., Gazy I., Jackson L., Hwa S.H., Tegally H., Lustig G., Giandhari J., Pillay S., Wilkinson E., Naidoo Y. (2021). Escape of SARS-CoV-2 501Y.V2 from neutralization by convalescent plasma. Nature.

[68] Madhi S.A., Baillie V., Cutland C.L., Voysey M., Koen A.L., Fairlie L., Padayachee S.D., Dheda K., Barnabas S.L., Bhorat Q.E. (2021). Efficacy of the ChAdOx1 nCoV-19 Covid-19 Vaccine against the B.1.351 Variant. N. Engl. J. Med..

[69] Mahase E. (2021). Covid-19: Novavax Vaccine Efficacy Is 86% against UK Variant and 60% against South African Variant. BMJ.

[70] Shinde V., Bhikha S., Hoosain Z., Archary M., Bhorat Q., Fairlie L., Lalloo U., Masilela M., Moodley D., Hanley S. (2021). Efficacy of NVX-CoV2373 Covid-19 Vaccine against the B.1.351 Variant. N. Engl. J. Med..

[71] Dejnirattisai W., Zhou D., Supasa P., Liu C., Mentzer A.J., Ginn H.M., Zhao Y., Duyvesteyn H.M.E., Tuekprakhon A., Nutalai R. (2021). Antibody evasion by the P.1 strain of SARS-CoV-2. Cell.

[72] Straten K., Guerra D., van Gils M.J., Bontjer I., Caniels T.G., van Willigen H.D.G., Wynberg E., Poniman M., Burger J.A., Bouhuijs J.H. (2022). Mapping the Antigenic Diversification of SARS-CoV-2. medRxiv.

[73] Suryawanshi R.K., Chen I.P., Ma T., Syed A.M., Brazer N., Saldhi P., Simoneau C.R., Ciling A., Khalid M.M., Sreekumar B. (2022). Limited Cross-Variant Immunity after Infection with the SARS-CoV-2 Omicron Variant Without Vaccination. medRxiv.

[74] Nyberg T., Ferguson N.M., Nash S.G., Webster H.H., Flaxman S., Andrews N., Hinsley W., Bernal J.L., Kall M., Bhatt S. (2022). Comparative analysis of the risks of hospitalisation and death associated with SARS-CoV-2 omicron (B.1.1.529) and delta (B.1.617.2) variants in England: A cohort study. Lancet.

[75] Simmonds P., Aiewsakun P., Katzouraki A. (2018). Prisoners of War—Host Adaptation and Its Constraints on Virus Evolution. Nat. Rev. Microbiol..

[76] Geoghegan J.L., Holmes E.C. (2018). The Phylogenomics of Evolving Virus Virulence. Nat. Rev. Genet..

[77] Bull J.J., Lauring A.S. (2014). Theory and Empiricism in Virulence Evolution. PLoS Pathog..

[78] GitHub, CSSEGISandData. https://github.com/CSSEGISandData/COVID-19/tree/master/csse_covid_19_data.

[79] Bernhauerová V. (2022). Adapting to Vaccination. Nat. Ecol. Evol..

[80] Paules C.I., Marston H.D., Fauci A.S. (2020). Coronavirus Infections-More Than Just the Common Cold. JAMA.

[81] Sun X., Wang T., Cai D., Hu Z., Chen J., Liao H., Zhi L., Wei H., Zhang Z., Qiu Y. (2020). Cytokine storm intervention in the early stages of COVID-19 pneumonia. Cytokine Growth Factor Rev..

[82] Kovacech B., Fialova L., Filipcik P., Skrabana R., Zilkova M., Paulenka-Ivanovova N., Kovac A., Palova D., Rolkova G.P., Tomkova K. (2022). Monoclonal antibodies targeting two immunodominant epitopes on the Spike protein neutralize emerging SARS-CoV-2 variants of concern. EBioMedicine.

[83] Markov P.V., Katzourakis A., Stilianakis N.I. (2022). Antigenic evolution will lead to new SARS-CoV-2 variants with unpredictable severity. Nat. Rev. Microbiol..

[84] Bull J.J., Rustom A. (2022). Which ‘Imperfect Vaccines’ Encourage the Evolution of Higher Virulence?. Evol. Med. Public Health.

[85] He C., He X., Yang J., Lei H., Hong W., Song X., Yang L., Li J., Wang W., Shen G. (2022). Spike Protein of SARS-CoV-2 Omicron (B.1.1.529) Variant Have a Reduced Ability to Induce the Immune Response. Signal Transduct. Target. Ther..

[86] Rössler A., Riepler L., Bante D., von Laer D., Kimpel J. (2022). SARS-CoV-2 Omicron Variant Neutralization in Serum from Vaccinated and Convalescent Persons. N. Engl. J. Med..

[87] Mallory R., Formica N., Pfeiffer S., Wilkinson B., Marcheschi A., Albert G., McFall H., Robinson M., Plested J.S., Zhu M. (2021). Immunogenicity and Safety Following a Homologous Booster Dose of a SARS-CoV-2 Recombinant Spike Protein Vaccine (NVX-CoV2373): A Phase 2 Randomized Placebo-Controlled Trial. medRxiv.

[88] Yu X., Wei D., Xu W., Li Y., Li X., Zhang X., Qu J., Yang Z., Chen E. (2022). Reduced sensitivity of SARS-CoV-2 Omicron variant to antibody neutralization elicited by booster vaccination. Cell Discov..

[89] Haveri A., Solastie A., Ekström N., Österlund P., Nohynek H., Nieminen T., Palmu A.A., Melin M. (2022). Neutralizing antibodies to SARS-CoV-2 Omicron variant after third mRNA vaccination in health care workers and elderly subjects. Eur. J. Immunol..

[90] Gruell H., Vanshylla K., Tober-Lau P., Hillus D., Schommers P., Lehmann C., Kurth F., Sander L.E., Klein F. (2022). MRNA Booster Immunization Elicits Potent Neutralizing Serum Activity against the SARS-CoV-2 Omicron Variant. Nat. Med..

[91] Nemet I., Kliker L., Lustig Y., Zuckerman N., Erster O., Cohen C., Kreiss Y., Alroy-Preis S., Regev-Yochay G., Mendelson E. (2022). Third BNT162b2 Vaccination Neutralization of SARS-CoV-2 Omicron Infection. N. Engl. J. Med..

[92] Bar-On Y.M., Goldberg Y., Mandel M., Bodenheimer O., Amir O., Freedman L., Alroy-Preis S., Ash N., Huppert A., Milo R. (2022). Protection by a Fourth Dose of BNT162b2 against Omicron in Israel. N. Engl. J. Med..

[93] Lauring A.S., Tenforde M.W., Chappell J.D., Gaglani M., Ginde A.A., McNeal T., Ghamande S., Douin D.J., Talbot H.K., Casey J.D. (2022). Clinical severity of, and effectiveness of mRNA vaccines against, covid-19 from omicron, delta, and alpha SARS-CoV-2 variants in the United States: Prospective observational study. BMJ.

[94] Choi A., Koch M., Wu K., Chu L., Ma L., Hill A., Nunna N., Huang W., Oestreicher J., Colpitts T. (2021). Safety and immunogenicity of SARS-CoV-2 variant mRNA vaccine boosters in healthy adults: An interim analysis. Nat. Med..

[95] Massare M.J., Patel N., Zhou B., Maciejewski S., Flores R., Guebre-Xabier M., Tian J., Portnoff A.D., Fries L., Shinde V. (2021). Combination Respiratory Vaccine Containing Recombinant SARS-CoV-2 Spike and Quadrivalent Seasonal Influenza Hemagglutinin Nanoparticles with Matrix-M Adjuvant. bioRxiv.

